# Why Do Farmers Not Irrigate All the Areas Equipped for Irrigation? Lessons from Southern Africa

**DOI:** 10.3390/agriculture14081218

**Published:** 2024-07-24

**Authors:** Luxon Nhamo, Sylvester Mpandeli, Stanley Liphadzi, Tinashe Lindel Dirwai, Hillary Mugiyo, Aidan Senzanje, Bruce A Lankford, Tafadzwanashe Mabhaudhi

**Affiliations:** 1 Water Research Commission of South Africa, Lynwood Manor, Pretoria 0081, South Africa; 2 Centre for Transformative Agricultural and Food Systems (CTAFS), School of Agricultural, Earth and Environmental Sciences, University of KwaZulu-Natal, Pietermaritzburg 3209, South Africa; 3 Faculty of Science, Engineering and Agriculture, University of Venda, Thohoyandou 0950, South Africa; 4 Department of Environmental, Water and Earth Sciences, Tshwane University of Technology (TUT), Pretoria 0029, South Africa; 5 International Water Management Institute (IWMI), P.O. Box MP163, Mount Pleasant, Harare, Zimbabwe; 6 Ministry Lands, Agriculture, Water, Fisheries and Rural Development, 1 Borrowdale Road, P.O. Box MP163, Mount Pleasant, Harare, Zimbabwe; 7 School of Engineering, University of KwaZulu-Natal, Pietermaritzburg 3209, South Africa; 8 School of Global Development, University of East Anglia, Norwich NR4 7TJ, UK; 9 Centre on Climate Change and Planetary Health, London School of Hygiene and Tropical Medicine (LSHTM), London WC1E 7HT, UK; 10 United Nations University Institute for Water, Environment and Health, Richmond Hill, ON L4B 3P4, Canada

**Keywords:** agricultural productivity, agriculture water management, food security, irrigation potential, land use policy

## Abstract

The reliance on rainfed agriculture exposes southern Africa to low agricultural productivity and food and nutritional insecurity; yet, the region is endowed with vast irrigation potential. Extreme weather events including drought, floods, and heatwaves exacerbate the existing challenges, underscoring the need to improve agricultural water management as a climate change adaptation strategy. This mixed-methods review followed the Search, Appraisal, Synthesis, and Analysis (SALSA) framework to explore the irrigation opportunities and challenges in southern Africa by critically analysing the drivers and constraints of irrigation systems in southern Africa. The premise is to understand the reasons behind the abandonment of some of the areas equipped for irrigation. In cases where irrigation systems are present, the study assesses whether such technologies are effectively being used to generate the expected agricultural productivity gains, and what factors, in cases where that is not the case, constrain farmers from fully using the existing infrastructure. The review further discusses the enabling environment supporting irrigated agriculture and the role of gender in irrigation development. An assessment of the role of women in agriculture on the share of land equipped for irrigation to total cultivated land area, as well as on the proportion of the area equipped for irrigation versus the area that is actually irrigated is conducted. The review found a divergence between countries’ land areas equipped for irrigation and actually irrigated areas. Specific to irrigation expansion, the review rebuts the notion that increasing the irrigated area increases crop production and ensures food security. This may not always be true as irrigation development needs to consider the impacts on other closely linked water and energy sectors through transformative approaches like the water–energy–food (WEF) nexus and scenario planning. If well-planned and implemented, sustainable irrigated agriculture could be catalytic to transforming southern Africa’s food system to be inclusive, equitable, socially just, and resilient, benefiting people and the planet.

## 1 Introduction

Southern Africa is a region of many challenges including low economic growth that is skewed towards the fortunate few, climate change extremes, food and water insecurity, high unemployment rates, and high poverty levels, among other challenges [1,2]. Rapid urbanisation and regional migration seldom improve the lives that people expect to be improved due to inequality, climate change, and the prevalence of diseases which continue to decimate the region and the lives of over 60% of the population that lives in rural areas depending on agricultural and natural resources for their livelihoods [3]. These challenges have entrapped the region into a vicious cycle of poverty, exposing the majority to vulnerabilities of various forms and magnitudes [4]. Vulnerability and low agriculture productivity present a tough combination for smallholder farmers who comprise most of the population as they rely on highly climate-sensitive rainfed agriculture [5]. Recent droughts which are manifesting with a high intensity and frequency compound this unfortunate situation. While the share of the expenditure on agriculture has increased towards the recommended 10% of the Gross Domestic Product (GDP) over the past decade in several countries, irrigation expansion remains generally low as the irrigated area remains below the regional target of 7% [6–8]. Although some research has pointed to the inter-temporal risks of under-investments in agriculture and water management, policy change towards more investment is slow for several reasons including weak country-level platforms that can catalyse change [9].

The need to tap into the vast irrigation potential of southern Africa is instigated by the pace at which the population is increasing (expected to reach 2.5 billion by 2050) [10], compounded by the increasing greenhouse gas (GHG) emissions and rising temperatures above pre-industrial levels [11]. There is pressure on agriculture to continue meeting the food requirements of the increasing population amidst climate change, and resource degradation and depletion. The challenges are having negative impacts on lives and livelihoods [12]. As a result, the region is forecasting a 15% decline in agricultural yields by 2050 if global warming increases by 2 °C [13], impacting the livelihoods of over 60% of the rural population who depend on agriculture. Therefore, the region would be able to meet only about 13% of its food requirements by 2050 [14,15]. Furthermore, between 350 and 600 million people in 2050 in southern Africa will be living in water-stressed areas [16]. The projections indicate a greater risk to lives and livelihoods, threatening human and environmental health, and risking sustainable development. There is, therefore, an urgent need to transition from the current rainfed and monoculture type of agriculture to a more viable and resource-efficient irrigated agriculture that ensures food and water security in the long run [17,18]. Southern Africa has been characterised by low rainfall and recurrent droughts since the early 1980s and 1990s, negatively impacting agricultural productivity in the region. For example, the region experienced the worst droughts in 1992 and 2015, resulting in smallholder farmers losing large herds of livestock and total crop failure due to low rainfall [4].

The highly variable and insufficient rain, which often results in increased incidences of droughts, implies that irrigation plays a critical role in agricultural productivity [5,19]. Yet, several parts of the region’s land equipped for irrigation are not actually irrigated [5,20]. In the whole of Africa, for example, most of the irrigated land is concentrated in five countries, mainly Egypt, Madagascar, Morocco, Sudan, and South Africa, occupying more than two-thirds of the existing irrigated area of the whole continent [21]. This is evidence that crop production in the continent is largely rainfed as indicated by the small share of the irrigated area in relation to the total cultivated area which is estimated at only 6% [5,20]. This is a relatively small fraction when compared to other regions like Asia with 37% and 14% for Latin America [20,22]. The underutilisation of the irrigation potential and the reliance on rainfed agriculture expose the continent to climate change risks [23].

This study explores the irrigation potential of southern Africa, highlighting the factors that explain the divergence between countries’ land areas equipped for irrigation versus the area that is actually irrigated. The study further examines the roles of water access and availability, land degradation, climate change, access to agricultural inputs and investment, and gender disparities on the proportion of the area equipped for irrigation that is actually irrigated. The premise was to understand why some areas equipped for irrigation are not being irrigated and to provide recommendations that could support the region in fully realising its irrigation potential and improving water and food security.

## 2 Methods

### 2.1 Data Collection and Analysis

This mixed-methods review used the simplistic Search, Appraisal, Synthesis, and Analysis (SALSA) framework [24] to search, extract, and analyse literature. The SALSA review method formed part of an integrated and transdisciplinary mixed approach as it included experts from various fields. The approach allowed for deriving coherent conclusions by using existing literature and databases from different sources. This was enhanced by data download from existing datasets and their analysis, review of existing literature, and expert knowledge on irrigation in southern Africa. The main tools used to achieve the objectives include Geographic Information Systems (GISs) to analyse and map irrigated and rainfed areas, water infrastructure, and the role of gender in irrigation. Therefore, the selection of a scoping review method as a key knowledge synthesis approach facilitated the identification of irrigation trends and gaps in southern Africa from an existing knowledge base based on existing literature and databases [25]. The review results were then used to inform research, policy, and practice.

Mixed-method reviews facilitate sensitive and separate search methods in different databases to retrieve relevant studies [24]. Therefore, the literature used was sourced from search engines including Web of Science (WoS), Scopus, and Google Scholar, from where 122 articles were accessed using search words like “irrigation in southern Africa”, “gender in irrigation”, “irrigation as a farming enterprise in developing countries”, “irrigation water infrastructure”, and “irrigation versus rainfed agriculture”, among other search words. However, the number of published articles eventually used came down to 86. Datasets from the Food and Agricultural Organisation’s (FAO) AQUASTAT [26] and the International Water Management Institute (IWMI) were used to complement the articles identified for literature synthesis, for example, generating the irrigated area map [27].

### 2.2 Literature Screening and Analysis Flowchart

**Figure 1:**
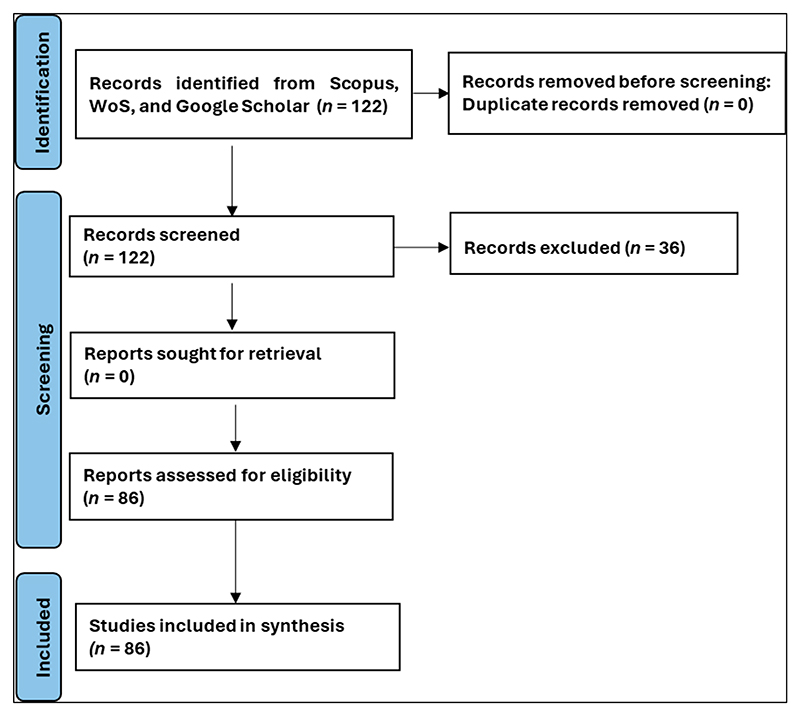
Schematic flowchart detailing the phases of literature search, handling, and screening.

Figure 1 is a schematic flowchart showing how the literature was handled and quantified. The collective search across trusted academic databases (Scopus and Google Scholar) and search platforms (WoS) yielded *n* = 122 articles, and, after the screening, *n* = 36 articles were excluded. This resulted in *n* = 86 articles that were utilised for synthesis. The literature (*n* = 86) was used to explore the causes of the underutilisation of irrigation potential in southern Africa. Southern Africa faces acute food and water insecurity, yet it has vast agricultural land under rainfed agriculture, and some of the cultivated land equipped for irrigation is not being irrigated [26,28,29]. The review, coupled with expert opinion and the authors’ knowledge of the region, identified four main contributing factors to the underutilisation of the irrigation potential in the region, including (a) lack of capacity for irrigation technologies and farming enterprise, (b) gender inequality in irrigation development, (c) lack of operational and management skills in irrigation development, and (d) unreliable energy sources. These factors formed the basis of the study and were used to meet the project objectives.

## 3 Results and Discussion

### 3.1 Irrigation Potential

**Figure 2:**
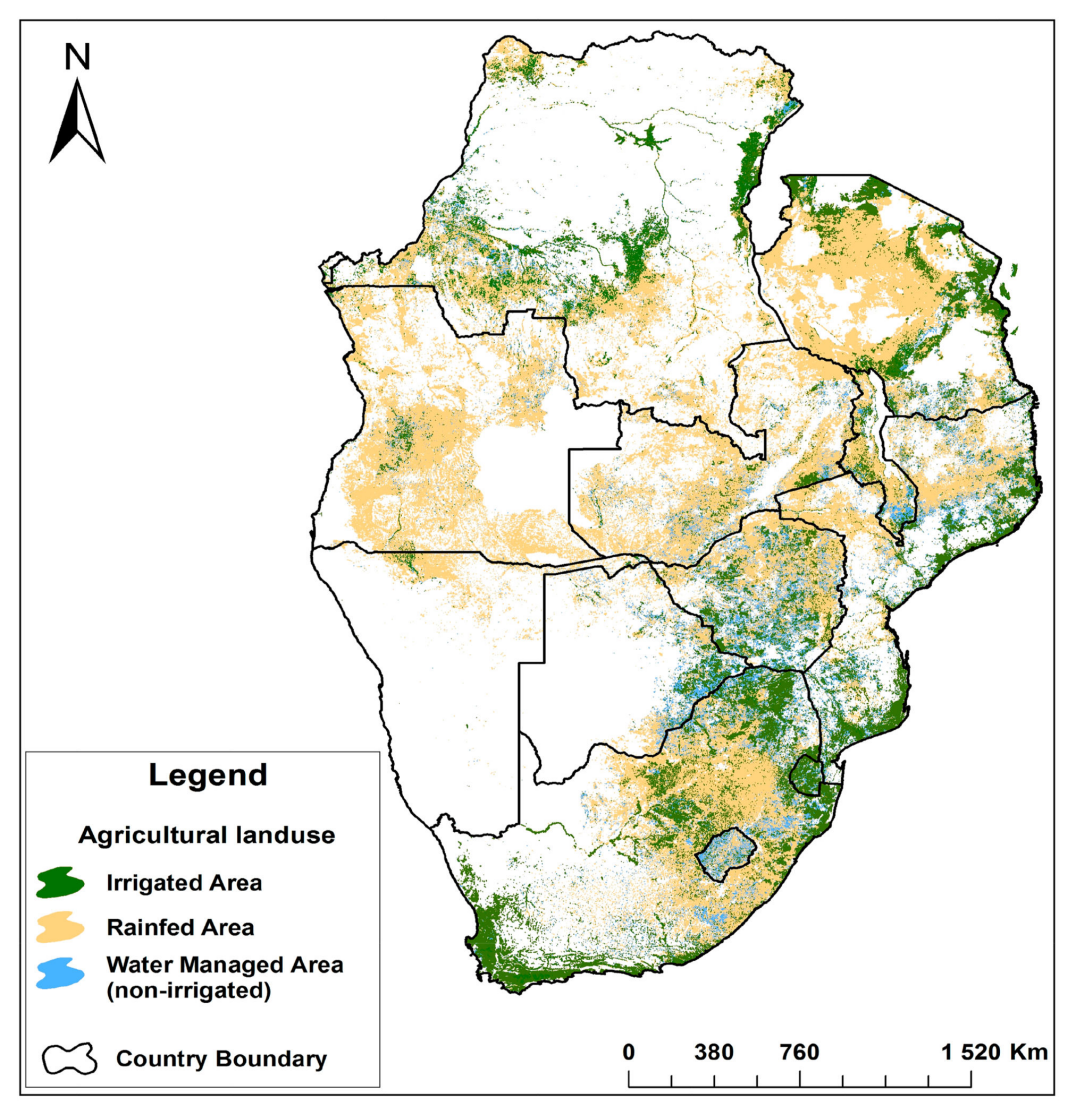
Share of irrigated and rainfed agricultural system in southern Africa. Source: Siddiqui et al., 2016 [27].

In Africa, cultivated land is estimated at around 1162 million hectares, which is about 40% of the continent’s total land area [30]. However, only 6% of this cultivated land is irrigated, a very low percentage compared to the world average of 18% [26,30]. In the case of southern Africa, most of the cultivated land is rainfed as only 9% of it is irrigated (Figure 2). Most regional countries have large tracts of land that are not irrigated, and some of the land that is equipped for irrigation is not being irrigated [20,27,31]. Except for South Africa which has an advanced irrigation sector, the rest of the region is not utilising its irrigation potential, contributing to low economic growth and chronic food and nutritional insecurity [20,32]. Yet, regional irrigation development can potentially increase crop production and productivity by 50% and subsequently improve the livelihoods of smallholder farmers who are generally vulnerable to climate change vagaries due to the reliance on rain agriculture [5,31].

The land with irrigation potential in southern Africa is about 40 million hectares, but only 17.5% is equipped for irrigation. Of this 17.5% (7 million hectares) of land that has irrigation potential, 20% is not irrigated [26,30]. This indicates that some of the irrigation equipment is not being used; therefore, the full benefits of irrigation have yet to be realised. This highlights the absence of efforts to enhance the youth’s skills and knowledge in irrigation and agriculture [29,33]. The need for capacity development in the irrigation subsector in the region is huge [34], particularly in novel irrigation technologies [35]. Human capacity development in agriculture should be accompanied by investment supported by strong institutions to drive the Fourth Industrial Revolution (4IR) in irrigation [36,37]. Smallholder farmers need to be exposed to technologies or innovations that will assist them in improving their income, increasing production, saving water and energy, etc. There is a high probability that such technologies will be adopted quickly across the region.

### 3.2 Dam Storage Potential

**Figure 3:**
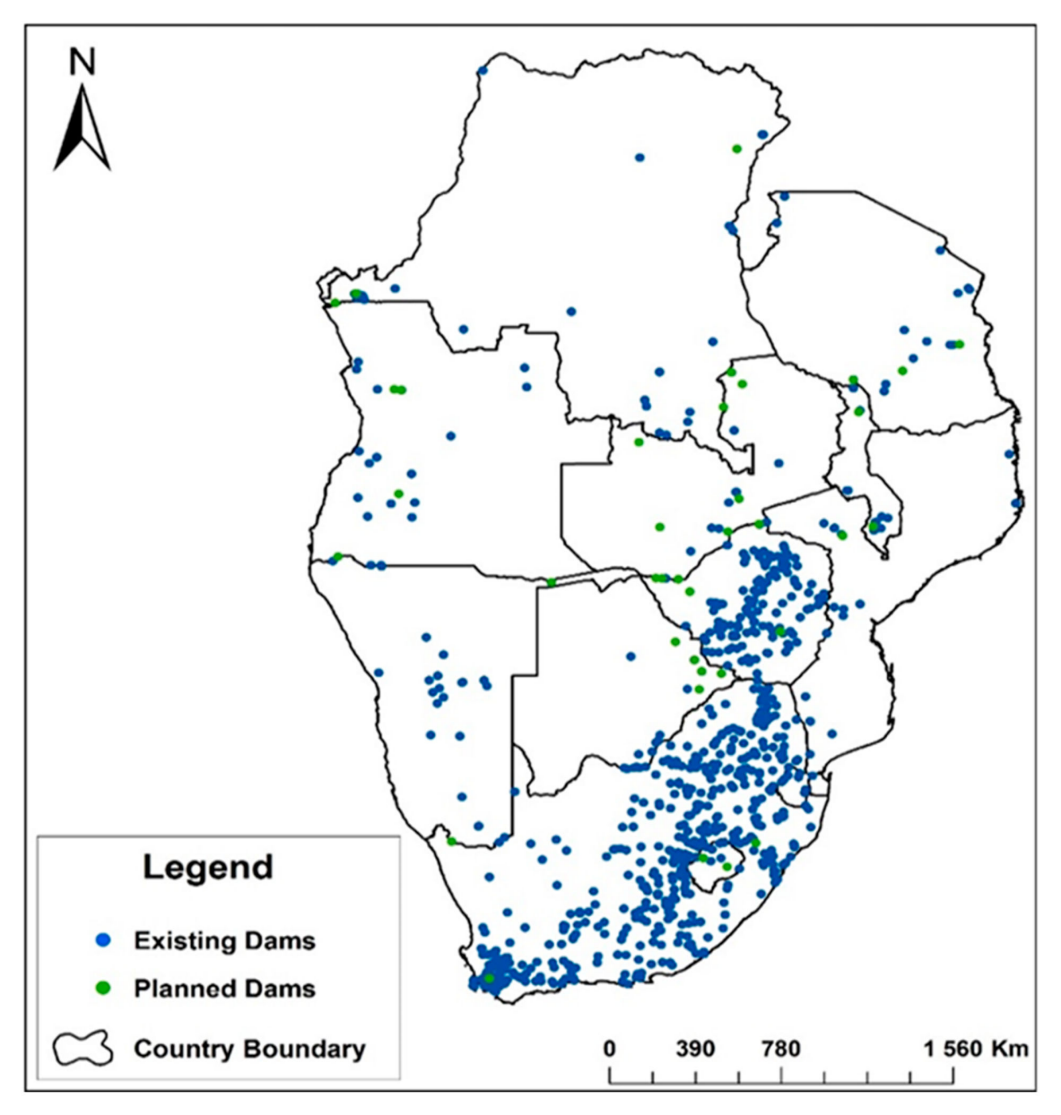
Existing and proposed dams in southern Africa indicating the uneven distribution across the region. Source: Data obtained from FAO AQUASTAT, 2024 [26].

Southern Africa receives a total mean annual runoff (MAR) of about 98 926 km^3^/year, but only 0.43% is harvested for future use, while 99.57% flows freely to the oceans [5,26]. Adding more surface water reservoirs to the existing ones is a prerequisite for irrigation development during drought periods and during the winter dry season. Irrigation water availability throughout the year allows crop production during both the rain and dry seasons, improving crop production, creating employment opportunities, improving the livelihoods of smallholder farmers, and boosting the economy [27,38,39]. Except for South Africa and Zimbabwe where there is already a high density of dams (Figure 3) [26], other regional countries still have the space to build more dams. The current predominant rainfed agriculture is unreliable and vulnerable, exposing the region to poverty and food insecurity.

The damming of surface runoff is particularly relevant in southern Africa as projections indicate that about 14% more water will be needed for irrigated agriculture by 2030, requiring an extra 220 km^3^ of storage [40]. Recent studies also indicate that the storage capacity of existing dams is decreasing due to siltation, resulting in storage losses of about 1% or 60 km^3^ yr^−1^ [4,5,40]. This demands the construction of more reservoirs to meet the additional water requirements for the added irrigated area, whilst, at the same time, rehabilitating and maintaining the existing infrastructure [27,41]. Dam rehabilitation includes those in South Africa and Zimbabwe where there is a high density of dams (Figure 3) [26]. The need for dam construction in the region could be different for the Democratic Republic of Congo (DRC) as it has abundant natural water flows [42]. The abundant water resources in the DRC are capable of supplying the whole region with the resource as it is the most water-rich country in Africa, as more than 50% of the continent’s surface water and nearly a quarter of the continent’s internal renewable water resources are in the DRC [26]. Besides the abundant water resources in the DRC, most of the cultivated land is under rainfed agriculture (Figure 2).

Besides being overly dammed, South Africa and Zimbabwe also have much of their arable land equipped for irrigation (Table 1). However, the two countries have reached the limit of feasible dam construction [43], highlighting the need for regional water investment or water transfer strategies [44]. Adding the 38 planned dams in the northern countries may not be enough to meet future water needs, even for the energy generation needed to power the proposed 5 million hectares of land earmarked for irrigation by 2030 [40]. Therefore, increasing water reservoirs and the irrigated area should be informed by transformative, cross-sectoral, integrated, and holistic approaches including the water–energy–food (WEF) nexus and scenario planning that facilitate assessing the catchment-based water potential, improved water-use efficiency, and agricultural water management [5,45].

### 3.3 Gender and Irrigation Development

About 70% of the agricultural labour force in southern Africa is women, and about 80% of them work as food processors, which translates to two-thirds of the agricultural workforce [46]. Thus, women play a key role in agriculture in southern Africa as there are more economically active women in agriculture than men (Figure 3). The research indicates that, in areas with more economically active women in agriculture, there is more land that is irrigated, although it is less equipped for irrigation [33,47,48]. However, because of the female dominance in agriculture, there is insignificant investment and little agricultural technological development, and more of the land equipped for irrigation is not irrigated as a result of a patriarchal culture in the region [49]. Gender inequalities and low investment in women-dominated agricultural areas, coupled with the lack of governance frameworks and institutions that support women’s irrigation, are the major impeding factors to irrigation development in the region [47]. Although the majority of women are actively involved in agricultural activities, the majority of these women cannot make strategic decisions without consulting their husbands or male counterparts irrespective of whether the men are involved or not. If we want women to be at the forefront of farming, there is an urgent need to dismantle the patriarchal culture.

**Figure 4:**
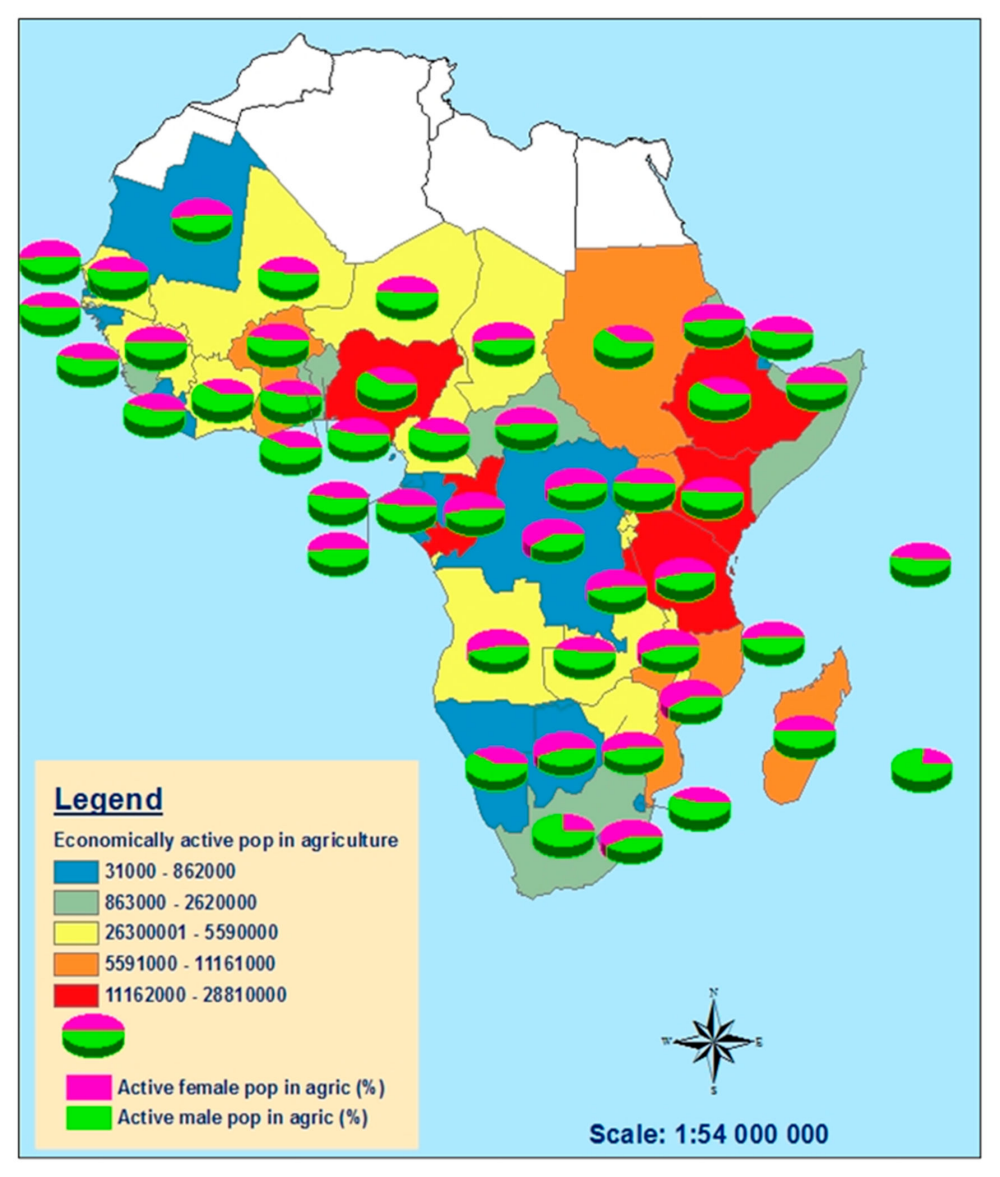
Economically active population in agriculture in Africa, which is predominantly women. Source: Data obtained from FAO AQUASTAT, 2024 [26].

Gender disparities in agriculture are evident in Lesotho, Mozambique, Zimbabwe, Botswana, and Tanzania. In contrast, in South Africa, Sudan, and Mauritius, where agriculture is predominantly carried out by men, there is considerable agricultural technology development and most of the land equipped for irrigation is actually irrigated (Figure 4). The lack of support for women in agriculture has slowed the economic development in the region. Overall, water resources in the region are unevenly distributed over a wide range of agro-ecological zones [44]. Currently, surface water remains the main source of irrigation water, while groundwater remains untapped [20,50]. The underutilisation of groundwater is mainly due to gender inequalities as women-dominated agriculture does not attract much investment [50]. As a result, only 2% of the region’s total renewable water resources are withdrawn annually for agricultural, industrial, and municipal purposes, meaning that 98% is not used [26]. Yet, agriculture sustains the economies of most countries in the region and the irrigation sector accounts for most of the freshwater withdrawals [2]. This indicates the underutilisation of the irrigation potential in the region; yet, more than 60% of the predominantly female population living in rural areas depends on rainfed agriculture for their livelihoods [3,51,52].

The role of women in agricultural development and food security cannot be overemphasised, yet it is largely unappreciated, including in policy that tends to be silent on the importance of women in crop production [53]. The realisation of the full irrigation potential in southern Africa needs to be backed by policies supporting and recognising the role of women in the sector and encouraging investment in women’s agriculture, including promoting access to credit and skills development in novel technologies targeted at women [54]. Although there are improvements in some countries, the role of rural women in decision-making processes generally remains obscure [55]. As highlighted several times, women do most of the work in agriculture; men still own the land, control women’s labour, and make agricultural decisions as patriarchal social systems are still very strong [56].

Research has shown that providing women with the same access as men to agricultural and land resources could increase crop production in women-led farms by 20 to 30% [57,58]. This could improve the total agricultural production from the current 2.5% to 4%, with the potential to reduce hunger by between 12 and 17%, or 100 and 150 million people [57]. Thus, agricultural production will increase if women in rural areas are capacitated with the right skills and given the same access to land, technology, credit services, and markets as men [28].

### 3.4 Policy Frameworks on Irrigation Development

Irrigation is key to increasing crop production and productivity, enhancing water and food security, and promoting agricultural sustainability and economic development [59]. Yet, in southern Africa, only 6% of the cultivated land is irrigated (13 million hectares), producing over 50% of crops grown [60]. Moreover, irrigation is regarded as a climate change adaptation strategy benefiting mostly smallholder farmers who contribute over 90% of the agricultural produce in southern Africa [54,61]. Based on the prospects of improving crop production through irrigated agriculture and improving economic growth in Africa, the African Union (AU) and regional economic communities have earmarked increasing the area under irrigation and supporting the resilience and sustainability of agriculture [62,63].

The transition from a rainfed-based system to an irrigated-focused one has seen a change in national, regional, and continental developmental goals in the African continent towards increasing investment for irrigation development [2]. The formulation of national and regional irrigation strategies is informed by continental and international strategies like the AU Agenda 2063, the AU Irrigation Development and Agricultural Water Management Framework (AU-IDAWM), and the United Nations’ 2030 SGDs, two frameworks that aim to reduce unemployment, hunger, and poverty by investing in agriculture [64,65]. The Agenda 2063 informed the prioritisation of growing African economies around agricultural growth and transformation through the 2014 Malabo Declaration [63]. These initiatives were followed by many other strategic frameworks and declarations promoting the adoption of sustainable irrigation and its rapid development, as well as agricultural water management, particularly smallholder irrigation development. These strategic frameworks include the Strategic and Operational Plan, 2014–2017, Fostering the African Agenda on Agricultural Growth and Transformation and Sound Environmental Management, AU/DREA January 2014) and Regional Economic Communities (RECs: IGAD, and ECOWAS, among others) in the National Investment Plans (NIP) and the National Agricultural Investment Plans (NAIP). The frameworks support the high-level political and strategic will on agriculture development as highlighted in the Comprehensive African Agriculture Development Program (CAADP) of the New Partnership for Africa’s Development (NEPAD) [62]. Pillar 1 of the CAADP emphasises land and water management, and irrigation development is well-highlighted [62]. Recently, the AU released the Framework for Irrigation Development and Agricultural Water Management (IDAWM) which was conceived against the backdrop of increasing climatic extremes that are causing reductions in agricultural production and impacting the livelihood capacities of rainfed agriculture in the continent [8].

Besides the presence of well-crafted policy frameworks, implementation at the national level has been slow, resulting in irrigation development remaining low at 6% since 2010, threatening food security on the whole African continent [66]. There is a need to translate the frameworks into practice and formulate a viable monitoring and evaluation framework [4].

## 4 Causes for Not Irrigating All the Land Equipped for Irrigation

As already alluded to, southern Africa faces chronic poverty and food insecurity, yet the region has a huge irrigation potential that needs to be fully utilised. Over 20% of the area equipped for irrigation is not irrigated, allowing the irrigation infrastructure to deteriorate as it lies idle [31,33]. The underutilisation of irrigation infrastructure is common in community-owned irrigation schemes [47]. There is generally a need for more management capacities as no one takes responsibility for repairing the infrastructure when broken, while the research reports infighting among the co-operative or community members [33,66]. This could highlight the poor management skills among the smallholder farmers. The challenges of poor management in irrigation schemes originate from the donor-backed formation of the schemes which did not involve the farmers themselves [31]. When the donors left, there was no financial backing to sustain the irrigation scheme enterprises resulting in their failure [67]. Moreover, during the irrigation modernisation era, non-contextual irrigation and hydraulic infrastructure were “imposed” on locals, thus creating a divide between functional and operational relationships between the hydraulic infrastructure and scheme governance [68]. The challenges are compounded by unsustainable land restitution programmes whose beneficiaries lack the expertise to manage large-scale irrigation enterprises [69,70].

Apart from the existing challenges in irrigation schemes, smallholder agriculture in the schemes remains subsistence where crops are grown on small family plots for household use and the little excess is sold to local communities [31]. The scope remains subsistence without the scope of venturing into commercial farming which opens access to external markets [33]. Thus, irrigation schemes have been converted to home food gardens where the produce is mainly for home consumption [71]. While this is important for household food security, rural livelihoods remain vulnerable and exposed to chronic poverty as agriculture remains subsistence. Moreover, subsistence agriculture is generally associated with poor farming methods that contribute to the degradation of cultivated lands [72,73]. Yet, peri-urban irrigation is market-oriented as the smallholder farmers produce perishable and high-value crops for the urban population where the extra money has transformed their livelihoods [74]. Thus, the lack of access to markets is a contributing factor to the underutilisation of the irrigation infrastructure, coupled with the unavailability of refrigerated storage facilities and poor transport networks to take the produce to distant markets [33]. The challenges have become a vicious cycle among smallholder farmers as they struggle to overcome the poverty trap without external support.

The other contributing factor to the underutilisation of the existing irrigation infrastructure is the lack of operational and management skills and maintenance expertise to repair the infrastructure among smallholder farmers [31]. As already alluded to, most of the existing infrastructure was put in place by donors or governments without investing in human resources, especially women who form the majority of people working in agriculture [33,58]. The lack of support for women-dominated agriculture contributes to the failure to fully realise the irrigation potential in southern Africa as women are still marginalised, lacking access to land and other resources like their male counterparts [75].

Furthermore, some areas equipped for irrigation are not irrigated due to a lack of energy to power the irrigation system. Poor off- and on-grid irrigation facilities in southern Africa contribute to the low irrigation development and the failure to irrigate some of the areas equipped for irrigation [34]. Although farmers revert to expensive fuel-based irrigation to compensate for the unavailability of the on-grid energy sources, this has been unsustainable due to the high costs involved, thus inhibiting irrigation expansion or even the abandonment of the existing irrigation infrastructure. Off-grid solar-powered irrigation has been shown to be a viable solution as farmers take advantage of the abundant solar energy resources in the region, overcoming many of the energy access constraints, but uptake has been slow [34,76] due to prohibitive establishment costs.

Land redistribution programmes being implemented across the region to correct historical and colonial land imbalances are also contributing to the underutilisation of irrigation infrastructure [77]. Although the initiatives could be noble, most of the beneficiaries lack the expertise to operate and maintain the irrigation equipment they inherit, resulting in a total collapse of the irrigation sector in particular and the whole of agriculture in general [78]. Land redistribution which does not consider the capacities of the beneficiaries and fails to consider holistic and cross-sectoral planning is bound to fail and exacerbate food insecurity [69].

The causes for not irrigating all the land equipped for irrigation can be grouped into four categories: (a) technical factors, (b) natural factors, (c) economic factors, and (d) administrative factors (Table 2). Technical factors include factors related to the lack of expertise in irrigation technologies and lack of interest in agriculture as a business [79]. These factors tend to drive the youth away from agriculture; yet, the sector can contribute to employment creation [80]. Natural factors refer to the occurrence of natural disasters that seem to be endemic in southern Africa, including pests and diseases, extreme weather events, and the lack of access to water and energy resources [2,4]. From an economic perspective, irrigation development in southern Africa is generally impacted by poor market access, high costs of labour and inputs, and high operational costs [20,31]. From an administrative point of view, there is a general lack of expertise from the locals, or the beneficiaries of the land redistribution programmes, poor irrigation management skills in operating irrigation co-operatives, and poor governance and institutional frameworks that support smallholder farming [29,31,69]. Table 2 provides the factors contributing to the underutilisation of the existing irrigation infrastructure and the possible solutions grouped in categories.

## 5 Recommendations

The current agricultural outlook in southern Africa, where the sector accounts for most of the freshwater withdrawals, yet food insecurity and poverty remain prevalent, requires immediate redress. Sustainable irrigation expansion and development are necessary, but they should not be realised in isolation from the other interlinked energy and water sectors. Irrigation expansion requires more energy to pump water resources from the source to the fields [81]. Water itself should be efficiently used as a scarce resource that needs to be shared with other sectors [5]. Therefore, focusing on irrigation expansion without considering synergies and trade-offs with the other linked sectors may end up in undesired outcomes including over-efficiencies in irrigation at the expense of water and energy sectors [5,45].

The water–energy–food (WEF) nexus could be adopted for appraising the potential benefits and/or implications derived from irrigation expansion initiatives [82], because, as a transformative approach, it establishes numeric relationships between WEF sectors to guide policy and decisions on development projects [45]. The WEF nexus integrative analytic model, for example, simplifies the human understanding of the interactions between the WEF resources, facilitating the identification of priority areas for interventions [45]. The interlinkages are expressed as interdependencies, constraints, and synergies that may manifest during the intervention, thus prompting timely corrective measures without compromising sustainability. Adopting the WEF nexus approach enhances resource use efficiency and sustainable development as developmental initiatives are not duplicated. Therefore, the approach is a strategic decision-support tool through which the resource development of interlinked sectors is undertaken [83].

As indicated, the irrigation potential remains untapped despite the rainfall in southern Africa being erratic and unreliable, and more than half of the economically active population is in agriculture. The inability to fully exploit the irrigation potential subjects the region to chronic poverty and economic depreciation. The region needs to consider the following to realise its irrigation potential fully:

- The reliance on rainfed agriculture increases vulnerability to food insecurity as crops are grown only during the rainy season. However, abundant groundwater resources can be drawn for irrigation during the dry winter or intra-seasonal drought periods. The main challenge of rainfed agriculture is that there is total crop failure when there is drought. Therefore, conjunctive water use in irrigation increases crop productivity, creates employment, reduces poverty, and enhances food and nutritional security.
- The current institutions must be strengthened through investment and supported by appropriate governance frameworks that guide and monitor the sector. Agricultural institutions should be established at both the national and regional levels, considering southern Africa’s transboundary nature of water resources. Currently, there are transboundary watercourse institutions that govern the transboundary water resources including the Zambezi Watercourse Commission (ZAMCOM), Limpopo Watercourse Commission (LIMCOM), and Orange-Senqu River Commission (ORASECOM), among others. The establishment of these institutions is quite important, but they need to be strengthened and work on developing policies that reduce the possibility of water conflicts but promote regional integration.
- The irrigation sector must gain the necessary skills to operate and maintain the irrigation technologies. As a result, some of the land equipped for irrigation is not being irrigated. The failure to fully exploit the irrigation potential and the underutilisation of the existing infrastructure in southern Africa is testimony to the scarce skills in the sector. There is, therefore, a need to develop the capacity in irrigation by establishing agricultural colleges to equip the farmers with the necessary skills including management and farm operation skills. This will go a long way toward transforming the irrigation sector into a fully operational and viable system as smallholder farmers take ownership of irrigation schemes. Training in agricultural skills is important in this era where 4IR and artificial intelligence (AI) are revolutionising agriculture.
- The 4IR coupled with the integration of novel technologies including AI, remote sensing, geographic information systems (GIS), blockchain, the Internet of Things (IoT), big data platforms, and drones, among other technologies, are transforming agriculture by facilitating smart irrigation and enhancing irrigation scheduling [84]. These intelligent technologies are being used to monitor soil moisture, predict weather conditions, adjust irrigation, and optimise irrigation scheduling, thus enhancing water use efficiency and crop-water productivity [36]. However, smart technologies require investment to fully realise the irrigation potential. This is important as climate change depletes freshwater resources and increases water scarcity.
- Therefore, the other critical area needing redress is more support and investment in woman-dominated irrigated areas. The lack of support for women in agriculture is resulting in women practicing agriculture on a part-time basis to supplement the income from their spouses who work in urban and mining areas. The policy should prioritise supporting women in agriculture as they dominate the agriculture sector. However, more investment and use of smart technologies will attract the youth to the sector which is currently dominated by women and the elderly [33].
- Equally critical is the need to address the co-operative nature of the existing irrigation schemes as most are lying idle due to the infighting of members and a lack of a sense of ownership of the irrigation infrastructure [31]. As a result, most of the irrigation schemes are abandoned, leaving the infrastructure to deteriorate [66]. Studies have shown that irrigation schemes have generally failed, calling for policymakers to come up with novel approaches to empower smallholder irrigation and revitalise irrigation schemes [31,33].
- As most irrigation schemes are being underutilised or have been abandoned completely, there is a need to formulate a strategy to revitalise the idle schemes [85]. The revitalisation efforts must include all stakeholders including farmers and extension officers. The revitalisation should be accompanied by improved institutions and investment opportunities. This is meant to recommend the appropriate irrigation technologies most suitable for smallholder irrigation schemes, including drip and micro-irrigation methods.

Overall, it is important to recognise the significance of irrigation in enhancing agricultural productivity gains and promoting economic growth for food security and livelihood improvement. However, these gains need to be supported and complimented by policies and institutions that are well-funded and run with the correct skills. Irrigation development also needs to be complemented by the right inputs and improved rural services including fertiliser availability, advanced seed delivery systems, post-harvest processing facilities, and access to markets [38]. As a climate change adaptation strategy, irrigation development has the advantage of doubling crop production and ensuring food and nutritional security, livelihood improvement, and economic growth.

The study demonstrates that sustainable irrigation through innovative technologies and practices is a climate change adaptation and resilience-building strategy, particularly in regions where the irrigation potential is not fully exploited. The reality is that water scarcity is increasing; at the same time, population growth and urbanisation are increasing, necessitating increased food production using less water, energy, and land, and without damaging the environment through greenhouse gas emissions [86]. This requires a paradigm shift to adopt systematic approaches that enhance resilience and adaptation through integrated actions, interventions, and investments [87,88].

The proposed recommendations are not peculiar to southern Africa alone but can be applied to other regions facing similar challenges and needing to increase the irrigated area. There is an urgent need for holistic and transformative interventions in agriculture to achieve sustainable food systems so that the sector may continue meeting the food demands of a growing population, at the same time without damaging the environment through greenhouse gas emissions [86]. Sustainable irrigation development is envisaged to provide cross-sectoral and equal weights to the environment and social and economic concerns, thus contributing to the security and health of people and the planet [69,89].

## 6 Conclusions

Irrigated agriculture has the potential to provide huge benefits, including income, employment, nutrition, and food security, by doubling the current agricultural productivity levels in southern Africa, where some of the land equipped for irrigation is not being irrigated. This study assessed the potential of southern Africa in increasing the irrigated areas and explored the causes of why some land equipped with irrigation infrastructure is not being irrigated. Irrigation development in southern Africa is complex, compounded by context-based challenges not easily addressed by traditional interventions, particularly irrigation schemes. The planned irrigation expansion in southern Africa by about 5 million hectares by 2030 has the potential to provide cumulative gains in terms of climate change adaptation, economic growth, poverty, unemployment, equity, and health outcomes. Nevertheless, the initiative is constrained by underlying challenges, including low investment, gender and land tenure imbalances, and lack of capacities, coupled with inequalities in social structures that manifest in institutions and markets due to inequalities in access to irrigation technologies and inputs. Therefore, gender inequalities in agriculture are a major constraint in irrigation development and the realisation of the full irrigation potential in the region. The study has shown that sustainable irrigation through innovative technologies and practices is important in enhancing climate change adaptation and building resilience. As water scarcity increases and rainfed agriculture becomes riskier, irrigation will play an increasingly larger role in securing not only food security but broader socio-economic and socio-ecological development. This study provides pathways to inform the transition from the current low-productive monoculture of rainfed agriculture to a more sustainable irrigated agriculture that is inclusive, equitable, socially just, and transformative, and does not exceed planetary boundaries. An integrated policy and institutional interventions that promote inclusive and sustainable farmer-led irrigation are key to irrigation development. The interventions should be guided and informed by transformative and circular approaches like the WEF nexus. A successful irrigation transformation should be accompanied by an investment in capacity development targeted to farmers, extension officers and a new generation of agricultural water managers, as the sector needs more relevant skills. The current priority of infrastructure support over skills and capacity development results in undesired outcomes.

## Figures and Tables

**Table 1:** Irrigation potential by country in southern Africa.

Country	Irrigation Potential (1000 ha)	Irrigation Potential Equipped for Irrigation (1000 ha)	Potential Area for New Irrigation Development (1000 ha)
Angola	3700	85.5	3614.50
Botswana	13	14	11.6
DRC	7000	10.5	6989.50
Lesotho	13	2.6	9.9
Madagascar	1517	1086.00	431
Malawi	162	73.5	88.4
Mauritius	33	21.2	11.8
Mozambique	3072	104.4	2967.60
Namibia	47	7.6	39.7
Seychelles	1	0.3	0.7
South Africa	1500	1500.00	0
Swaziland	93	49.9	43.4
Tanzania	2132	184.2	1947.80
Zambia	523	155.9	367.1
Zimbabwe	366	173.5	192.1
Southern Africa	20 172	6733.70	13 437.90

Source: FAO AQUASTAT, 2024 [25].

**Table 2:** Factors contributing to underutilisation of irrigation infrastructure and proposed solutions.

Category	Factors	Suggested Solutions
Technical factors	Lack of interest in agriculture	Invest in agriculture so that it becomes an attractive job creation sector. Promote novel technologies and attract the youth. Provide incentives to farmers.
	Poor knowledge of irrigation techniques and technologies	Establish dedicated agricultural colleges. Empower smallholder farmers through extension services. Initiate field-based training and research for smallholder farmers. Enhance extension services.
Natural factors	Pests and disease outbreaks	Develop early warning systems for pests and diseases. Promote a risk-based insurance policy for farmers.
	Insufficient water during the dry season	Tap into the groundwater resources. Introduce solar irrigation as a cheap energy source for irrigation.
	Soil failure on canal embarkments and landslides cause land degradation	Incorporate climate change in built environment for structural sound and adequate hydraulic infrastructure. Promote net zero land degradation approaches in vulnerable or disaster-prone areas.
Economic factors	Distance from the market	Build refrigerated storage facilities in smallholder farming areas.
	High costs of labour and inputs	Introduce agriculture mechanisation in smallholder farming areas. Subsidise agricultural inputs and technologies.
	Inability to access credit	Create affordable farmer-friendly credit lines from financial institutions. Incentivise agricultural inputs through support from the government.
	High operational and maintenance costs	Subsidise agricultural inputs and technologies. Establish dedicated agricultural colleges and enhance extension services.
Administrative factors	Lack of expertise	Establish dedicated agricultural colleges. Enhance extension services.
	In-fighting for control of irrigation schemes	Provide training to smallholder farmers. Introduce levies to irrigation scheme beneficiaries.
	Poor management of irrigation schemes	Provide training to smallholder farmers on farm management. Empower smallholder farmers through extension services.
	Poor governance and institutional frameworks	Formulate governance structures to support smallholder irrigation. Strengthen institutions that support smallholder farming.
